# Supplementary material for: Focused ultrasound excites cortical neurons via mechanosensitive calcium accumulation and ion channel amplification
Source: Nat Commun. 2022 Jan 25;13:493. doi: 10.1038/s41467-022-28040-1 (PMC8789820; doi:10.1038/s41467-022-28040-1)
Supplement: Supplementary file 1 — Supplementary Information [file 41467_2022_28040_MOESM1_ESM.pdf]

Supplementary Information

Focused ultrasound excites cortical neurons via mechanosensitive calcium accumulation and ion channel amplification

Sangjin Yoo[1], David R. Mittelstein[2], Robert Hurt[3], Jerome Lacroix[1,4], Mikhail G. Shapiro[1*]

[1]Division of Chemistry and Chemical Engineering, California Institute of Technology, Pasadena, CA 91125, USA
[2]Division of Engineering and Applied Science, California Institute of Technology, Pasadena, CA 91125, USA
[3]Division of Biology and Biological Engineering, California Institute of Technology, Pasadena, CA 91125, USA
[4]Graduate College of Biomedical Sciences, Western University of Health Sciences, 309 E. Second St, Pomona, CA 91766, USA
*Correspondence should be addressed to M.G.S. (mikhail@caltech.edu)

Supplementary Figures 1-11, Movie 1 and Tables 1-2

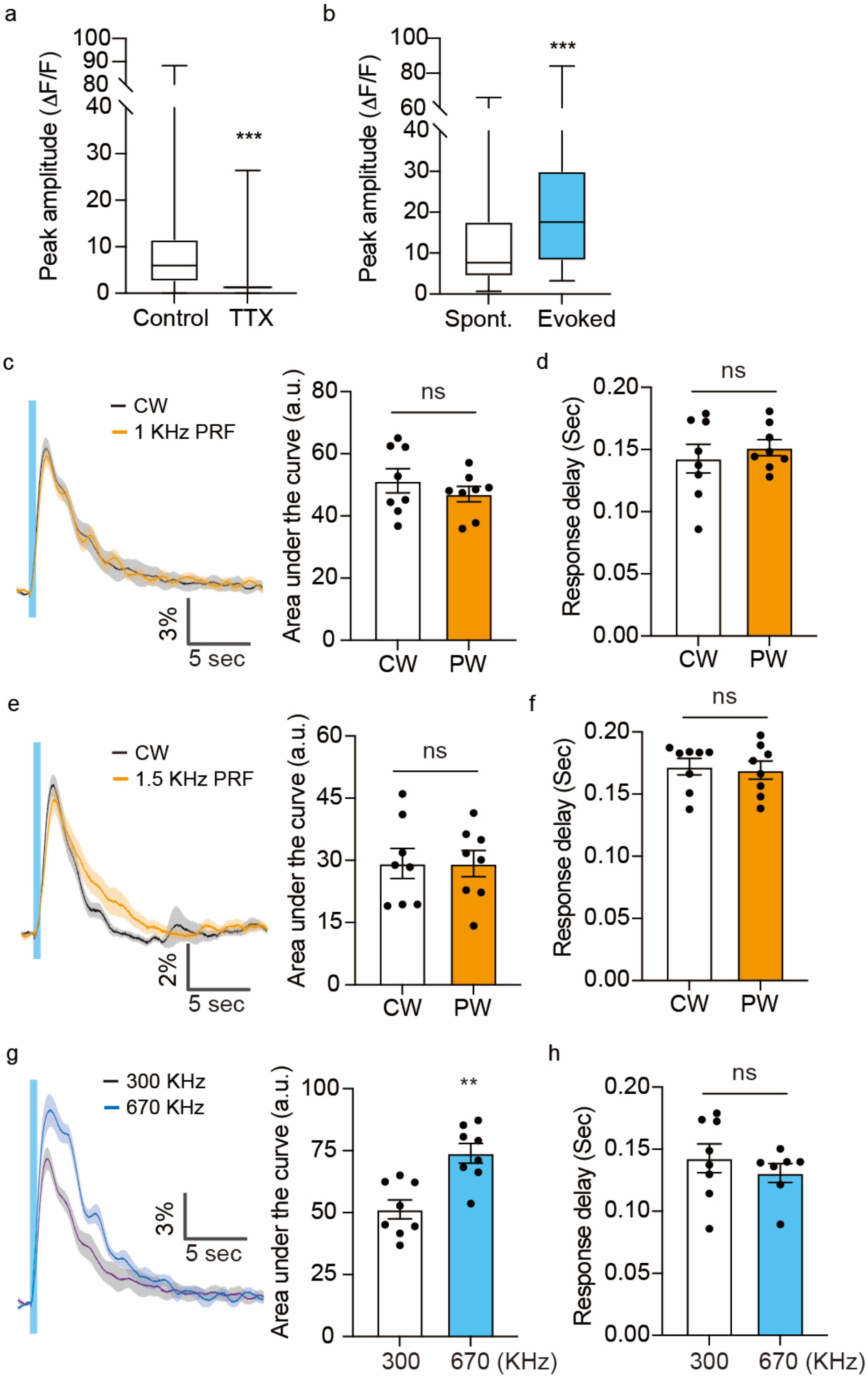

Supplementary Figure 1 | Neuron stimulation with pulsed-wave and higher frequency of ultrasound. (a) Comparison of peak amplitude of calcium response before and after TTX (Min/Max, Median within 50% volume, n= 370 (control), 429 (TTX), unpaired t-test, two-tailed, p<0.0001). (b) Comparison of peak amplitude of spontaneous calcium activity and evoked calcium response to ultrasound (Min/Max, Median within 50% volume, n=519, unpaired t-test, two-tailed, p<0.0001). (c) Comparison of calcium response to pulsed (1 KHz PRF) and continuous (CW) ultrasound (n=8 independent experiments, unpaired t-test, two-tailed, p=0.3667), and (d) their onset delay (n=8 independent experiments, unpaired t-test, two-tailed, p=0.5139). (e) Comparison of calcium response to pulsed (1.5 KHz PRF) and continuous (CW) ultrasound (n=8 independent experiments, unpaired t-test, two-tailed, p=0.9969), and (f) their onset delay (n=8 independent experiments, unpaired t-test, two-tailed, p=0.7882). (g) Comparison of calcium response with 300 and 670 KHz frequency of ultrasound (n=8 independent experiments, unpaired t-test, two-tailed, p=0.0011), and (h) their onset delay (n=8 independent experiments, unpaired t-test, two-tailed, p=0.4202). All values represent mean ± SEM.

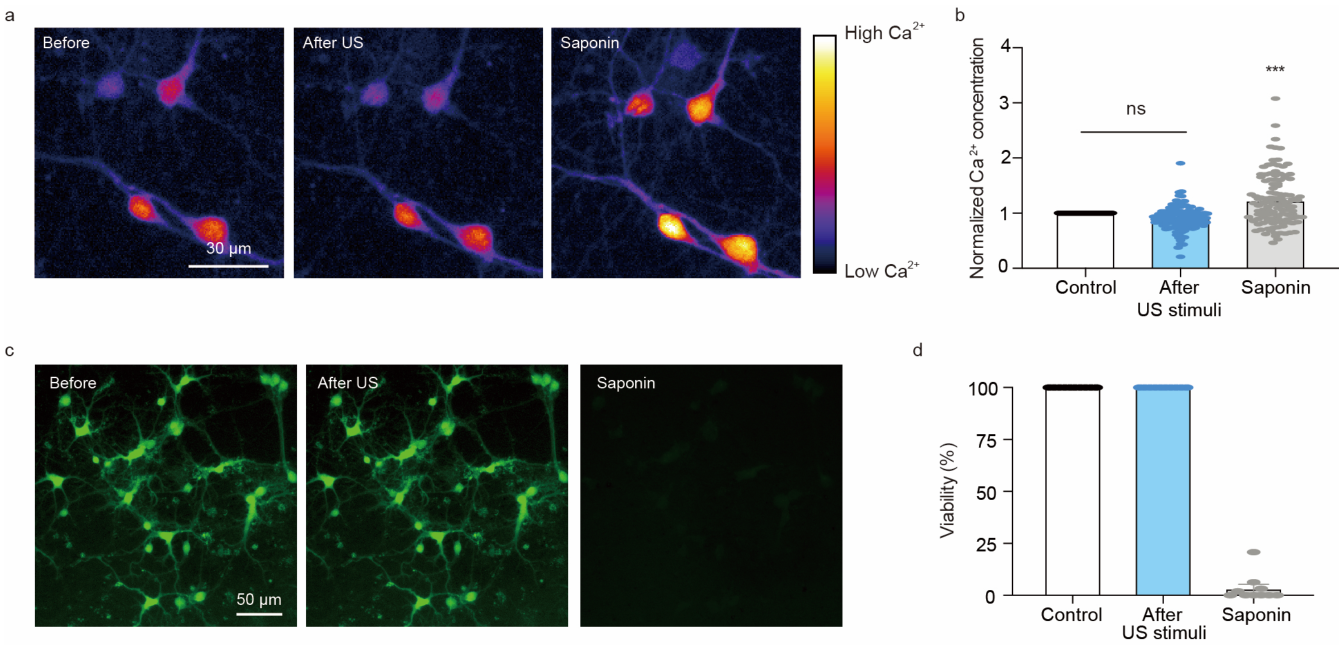

Supplementary Figure 2 | Safety test at cellular level after ultrasound stimulation. (a) Change of GCaMP6f baseline intensity before, after ultrasound stimuli (15 W/cm^2, 30 times every 20 sec) and after membrane poration (saponin), and (b) their quantification (n=117 cells from 2 dish, one way ANOVA followed by Tukey's multiple comparison test, $p=0.0587$ (control vs after US)). (c) Live neuron images before and after US stimulation and saponin treatment (after 5 mins from 100 µg/ml saponin treatment). And (d) quantification of neuron viability before and after the ultrasound stimuli and saponin treatment (n=10 ROI from 2 independent experiments). All values represent mean ± SEM.

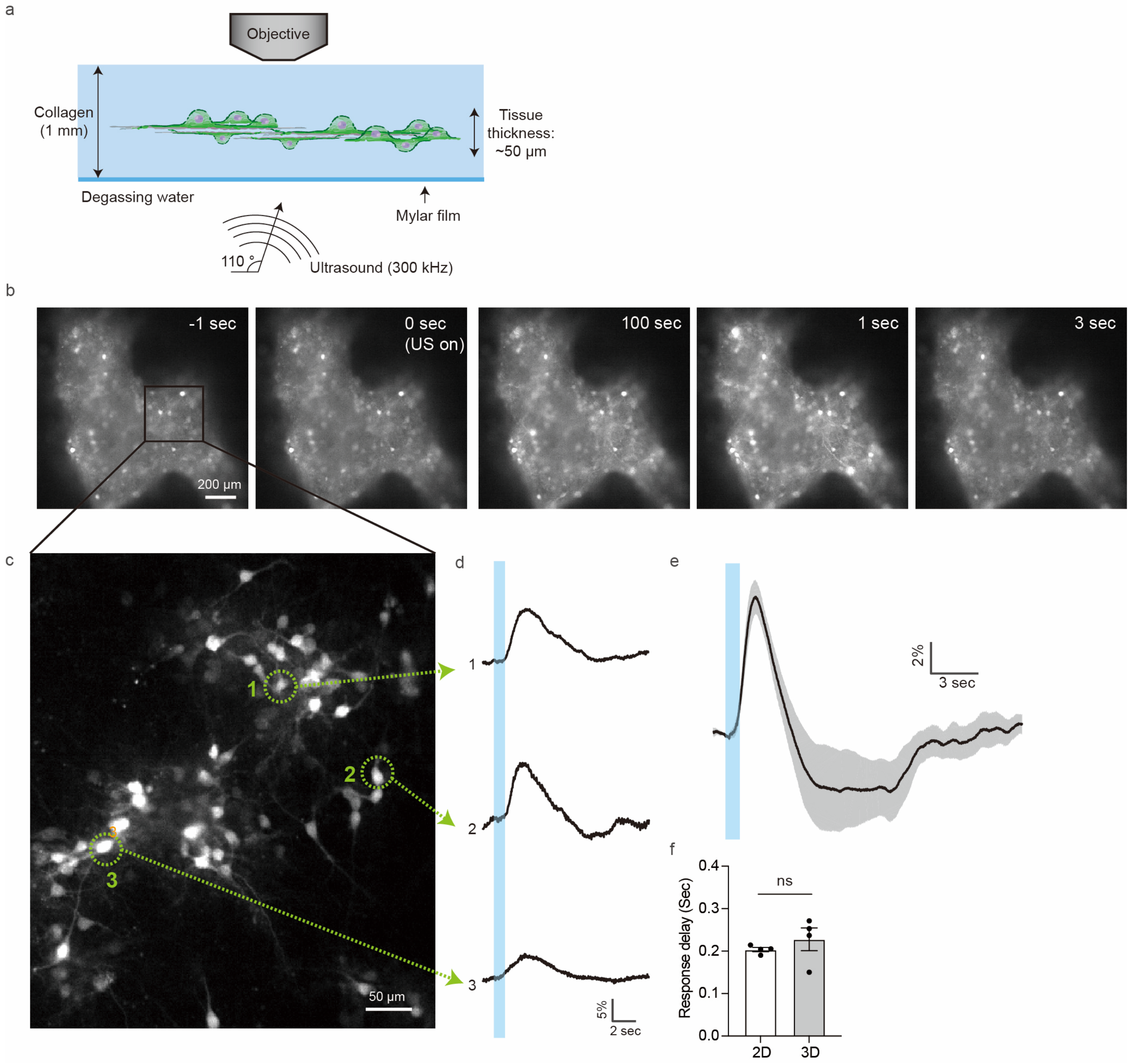

Supplementary Figure 3 | Ultrasound stimulation to 3D neural tissue model embedded in collage hydrogel. (a) Schematics of ultrasound stimulation to 3D collagen tissue model. (b) Time lapse images of calcium responses to ultrasound stimulation from the 3D cultured GCaMP6f neurons. (c) A snapshot of the 3D cultured GCaMP6f neurons and (d) Calcium responses from selected neurons. (e) Averaged calcium response (n= 4 independent experiments). (f) Quantification of response onset time (n= 4 independent experiments, Unpaired T-test, two-tailed, p= 0.4083). Mean trace is solid and SEM is shaded. All values represent mean ± SEM.

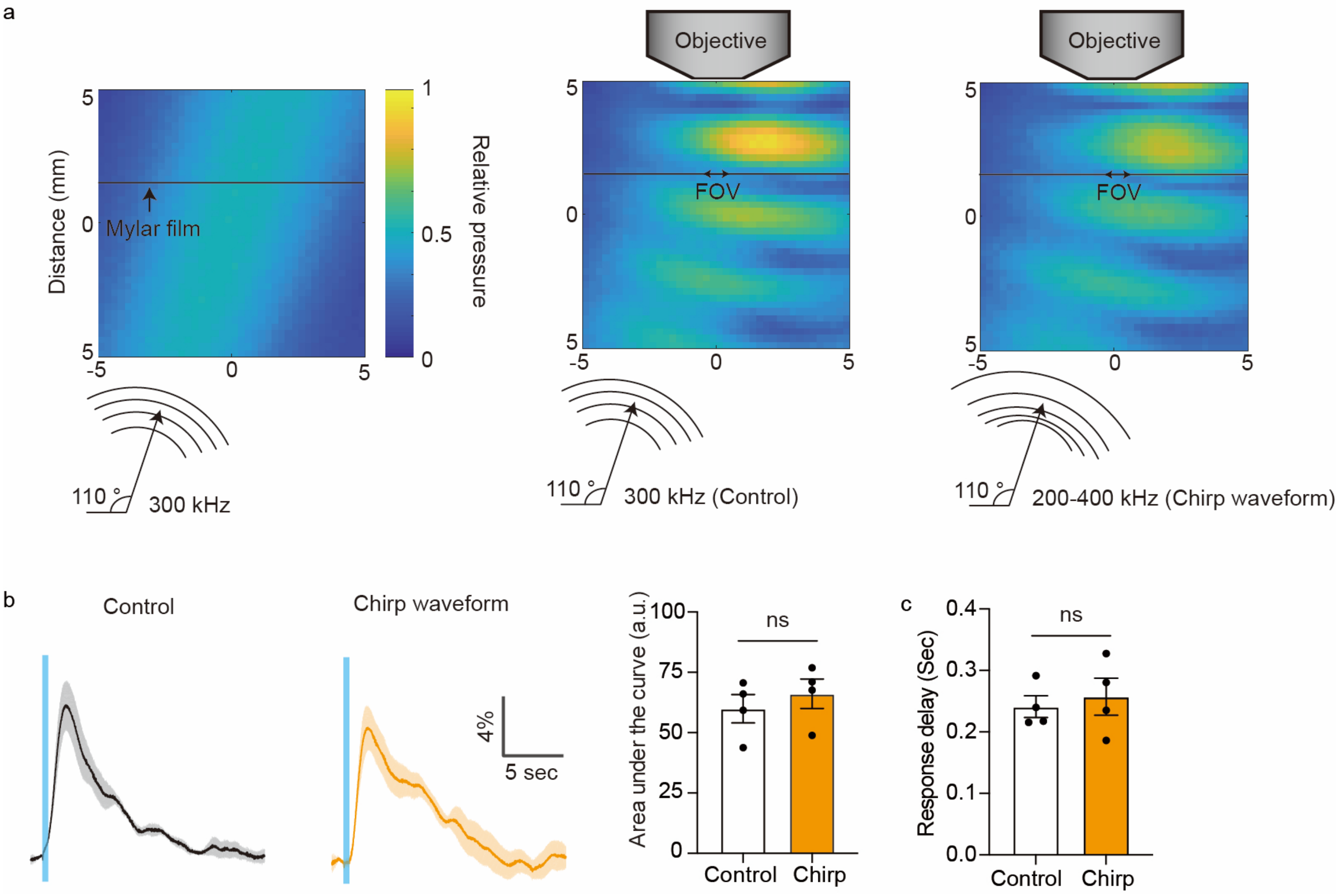

Supplementary Figure 4 | Ultrasonic neuron stimulation with chirp waveform. (a) Pressure profiles measured using a fiber optic hydrophone in three configurations. Left: deep underwater with no acoustic reflectors (i.e. "free field") with 300 kHz ultrasound. Middle: air/water interface and microscope objective lens at same locations as used in fluorescent imaging studies with 300 kHz ultrasound. Right: same configuration as middle with chirp waveform (Ultrasound frequency linearly increasing from 200 to 400 kHz over 10 μs, then linearly decreasing from 400 to 200 kHz over 10 μs in a repeating pattern throughout the 500ms burst). Standing wave pattern produced by reflection off objective lens decreased with chirp waveform compared to control waveform. (b) Calcium responses to ultrasound with normal and chirp waveform and their quantifications (n= 4 independent experiments, Paired T test, two-tailed, p=0.4891). (c) Quantification of response onset time (n= 4 independent experiments, Unpaired T-test, two-tailed, p=0.6591). Mean trace is solid and SEM is shaded. All values represent mean ± SEM.

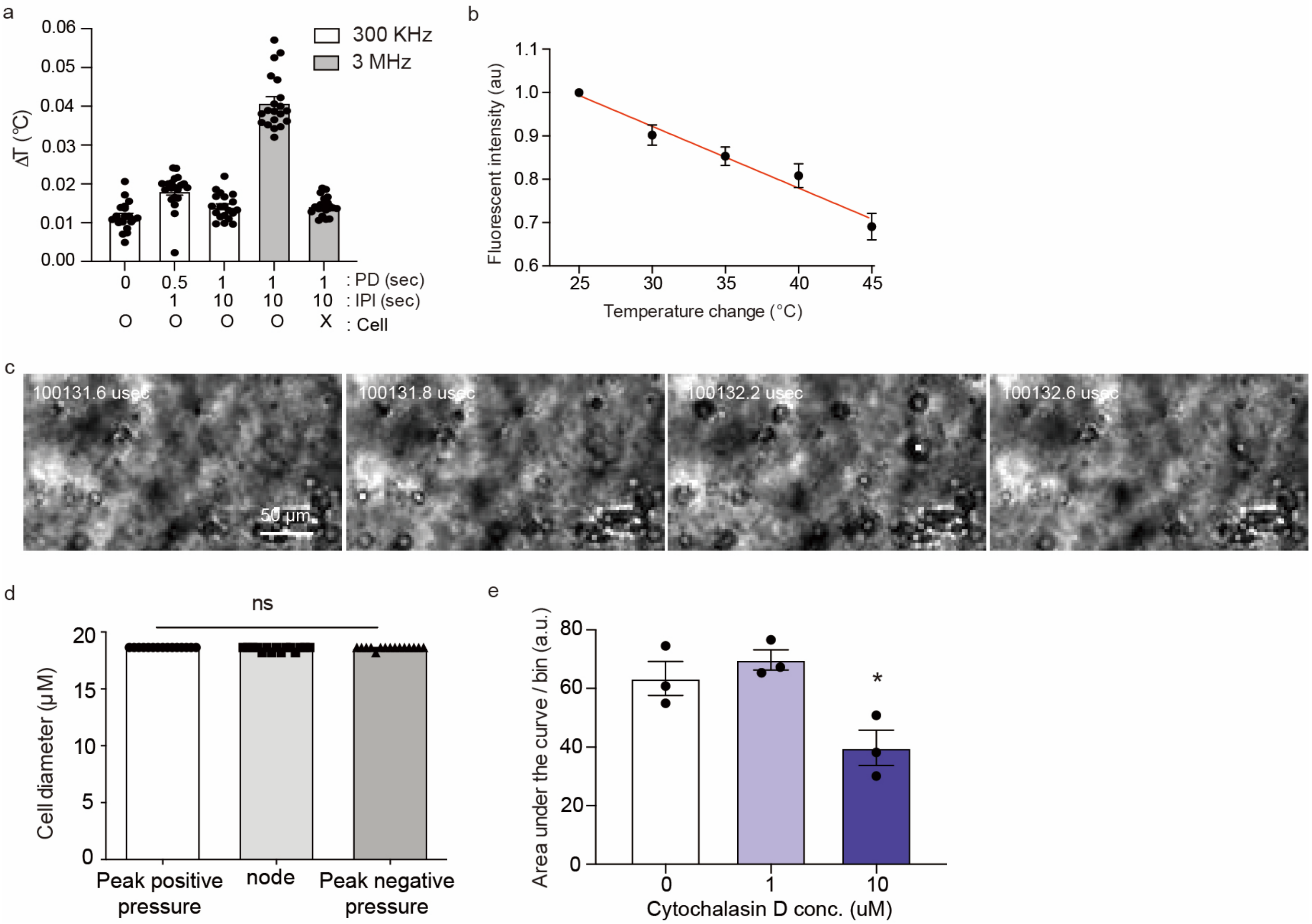

Supplementary Figure 5 | Ultra-high-speed imaging of neuron (a) Temperature measurement using an optic hydrophone near neurons during ultrasound stimulation with various parameters (n=20, 15 and 40 W/cm^2 intensities were used for 300 kHz and 3 MHz, respectively). (b) Fluorescence intensity of mCherry decreased as increasing the temperature (n=20, 2%/°C, R square=0.9240). (c) Ultra-high-speed imaging of microbubbles (bubble size=4 μm) while ultrasound stimulation. (d) Quantification of cell diameter change during ultrasound stimulation (n=15, Paired T test, two-tailed, p=0.3343). (e) Quantification of spontaneous activity before and after the actin depolymerization (bin =5 sec, n=3 independent experiments , Tukey's post comparison after one-way ANOVA, p=0.6859 (0 vs 1 μM)) with different concentrations of depolymerizers. All values represent mean ± SEM.

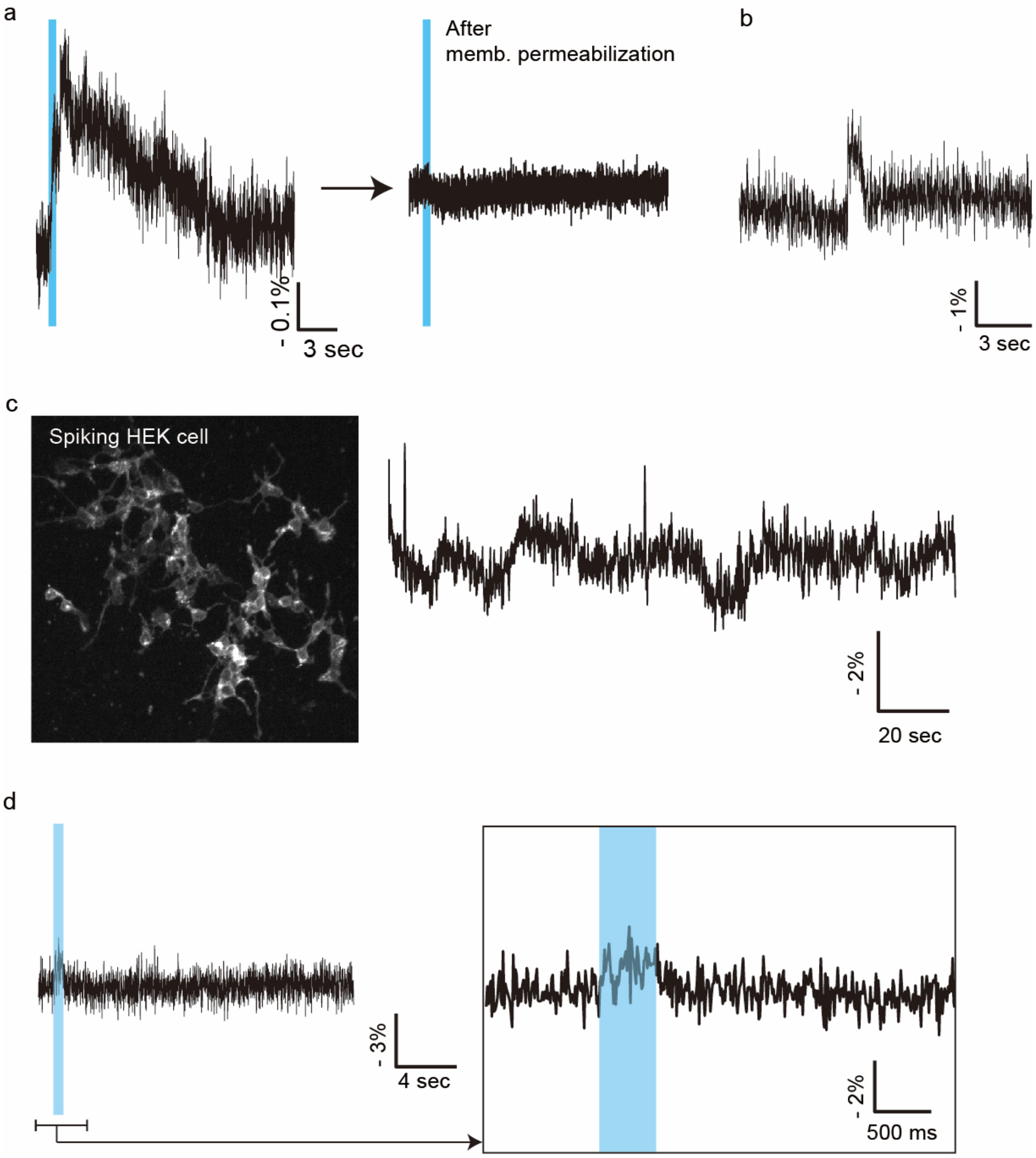

Supplementary Figure 6 | Voltage imaging of cells (a) Voltage responses to ultrasound (n= 4 independent experiments), and the responses disappeared after membrane permeabilization by saponin. (b) Spontaneous activity from Ace2N-expressing neurons in the absence of extracellular calcium. 1 μM bicuculine (GABAa blocker) was added to induce the hyper excitation (n=101 from a dish). (c) Ace2N-expressing spiking HEK cells and their spontaneous activity n=52 from a dish. (d) Voltage imaging of the spiking HEK cells during ultrasound stimulation, n= 82 cells from 2 independent experiments.

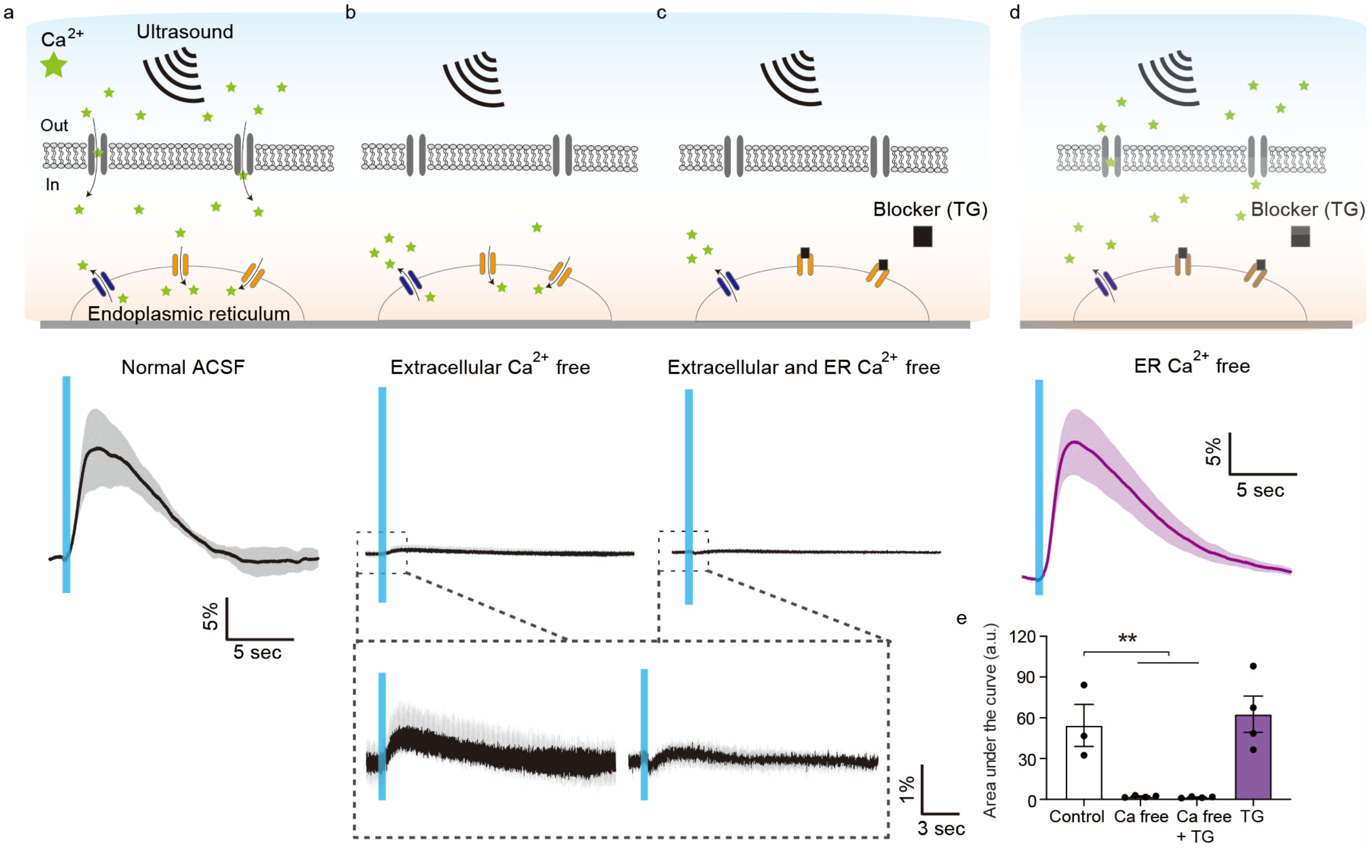

Supplementary Figure 7 | Ultrasound stimulation triggers calcium release from endoplasmic reticulum. (a) Calcium response to ultrasound stimulation in the presence of extra- and intercellular calcium (normal ACSF) (b) Calcium response to ultrasound stimulation in the absence of extracellular calcium (calcium free ACSF). (c) Calcium response to ultrasound stimulation in the absence of calcium in extracellular space and endoplasmic reticulum (calcium free ACSF + Thapsigargin). (d) Calcium response to ultrasound stimulation in the absence of calcium in endoplasmic reticulum (normal ACSF + Thapsigargin). (e) Quantification of area under the curve from each condition (n= 3 independent experiments for control, = 4 independent experiments for others, Unpaired t-test, two-tailed). Mean trace is solid and SEM is shaded. All values represent mean ± SEM.

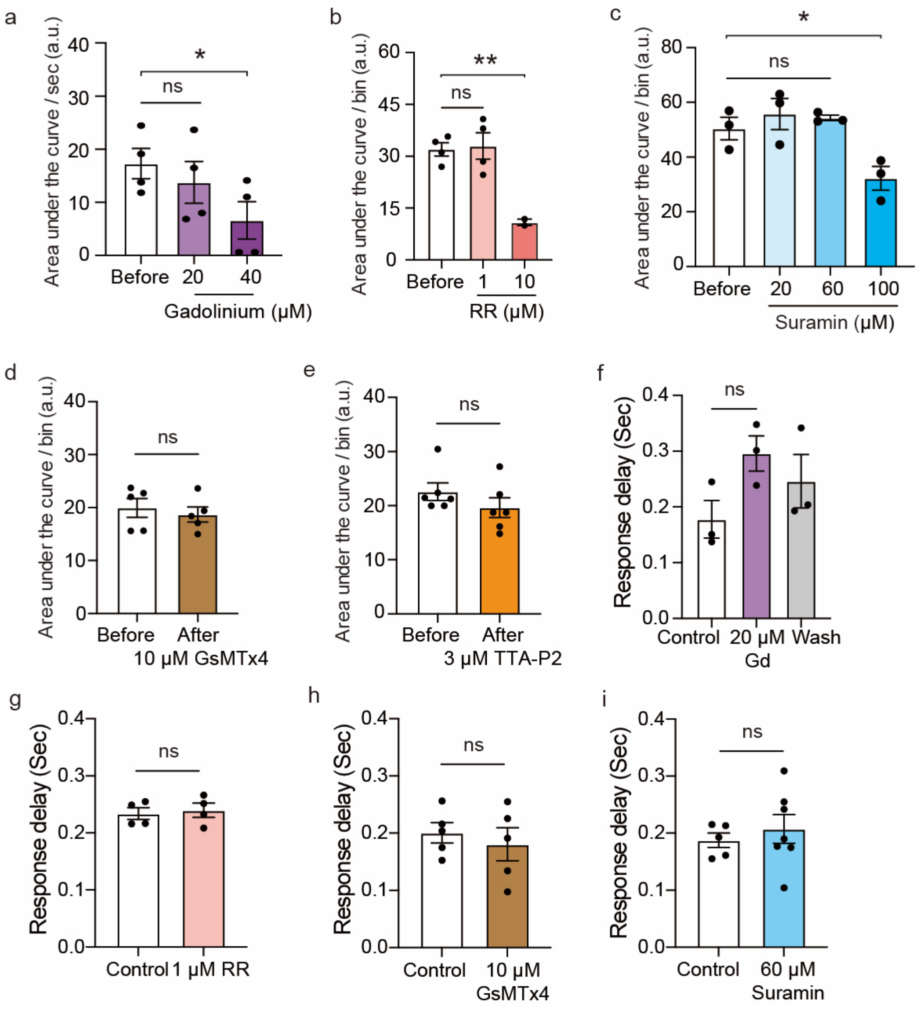

Supplementary Figure 8 | Spontaneous activity changes before and after channel blocking. (a) Spontaneous activity change after gadolinium treatment to block the global mechanosensitive channels (n= 4 independent experiments, Paired T-test, two-tailed, p=0.6258 (control vs 20 uM)). (b) Spontaneous activity change after ruthenium red treatment to block the TRPV1, 2 and 4 channels (n= 4 (1 uM) and 2 (10 uM) independent experiments , Paired T-test, two-tailed, p=0.8337). (c) Spontaneous activity change after suramin treatment to inhibit the GPCRs (n=3 independent experiments, Unpaired t-test, two-tailed, p=0.4167 (control vs 60 uM)). (d) Spontaneous activity change after GsMTx4 treatment to inhibit the Piezo1 and TRPC1 channels (n= 5 independent experiments, Unpaired t-test, two-tailed, p=0.6005). (e) Spontaneous activity change after TTA-P2 treatment to blocker the t-type calcium channels (n= 6 independent experiments, Paired T test, two-tailed, p=0.2546). Quantification of response onset time after (f) Gd^{3+} (n= 3 independent experiments, Tukey's multiple comparison after One-way ANOVA, p=0.1553 (Control vs. Gd)), (g) RR (n= 4 independent experiments, Unpaired T-test, two-tailed, p=0.7383), GsMTx4 (n= 5 independent experiments, Unpaired T-test, two-tailed, p=0.5719), Suramin treatment (n= 5 (control), 7 (suramin) independent experiments, Unpaired T-test, two-tailed, p=0.1183). All values represent mean ± SEM.

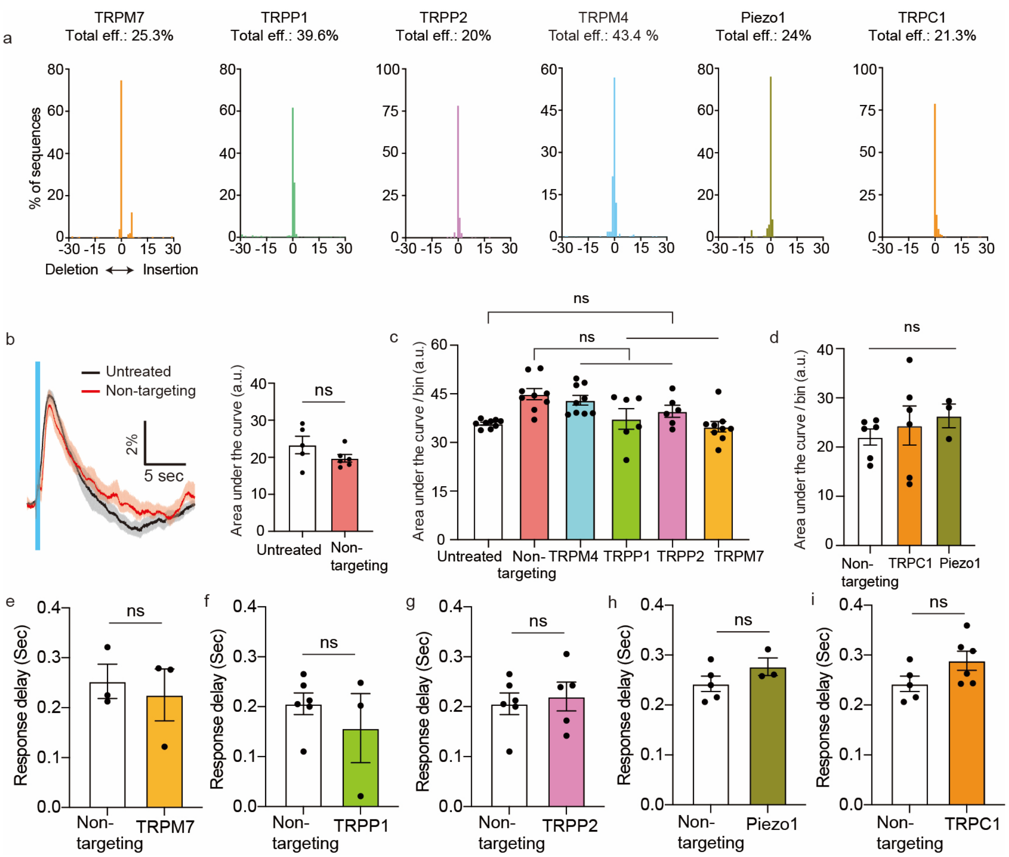

Supplementary Figure 9 | CRISPR/Cas9 for knockdown specific mechanosensitive ion channel. (a) CRISPR/Cas9 efficiencies for each targeted channel. (b) Calcium responses from wild type neurons and modified neurons (CRISPR/Cas9 with non-targeting sgRNA as a positive control (n= 5 independent experiments, Unpaired t-test, two-tailed, p=0.1720). (c and d) Quantification of spontaneous activities after knock down of the target channels (Dunn's multiple comparison after one-way ANOVA, n= 9 (Untreated, Non-targeting, TRPM4, TRPM7), 6 (TRPP1, TRPP2) independent experiments. p=0.0821~0.9999 (control vs channels), p=0.0024~0.9999 (Non-targeting vs channels), n= 6 independent experiments, p=0.8051~0.9999 (control vs channels)). Quantification of response onset time after CRISPR knockdown of (e) TRPM7 (n= 3, Unpaired T-test, two-tailed, p=0.6842), (f) TRPP1 (n= 6 (control), 3 (TRPP1), Unpaired T-test, two-tailed, p=0.4057), (g) TRPP2 (n= 6 (control), 5 (TRPP2), Unpaired T-test, two-tailed, p=0.6736), (h) Piezo1 (n= 5 (control), 3 (Piezo1), Unpaired T-test, two-tailed, p=0.2059), (i) TRPC1 (n= 5 (control), 6 (TRPC1), Unpaired T-test, two-tailed, p=0.1003), (j) TRPM4 (n=6 (control), 7 (TRPM4), Unpaired T-test, two-tailed, p=0.2332), and (k) after blocking T-type calcium channel (n= 5, Unpaired T-test, two-tailed, p=0.7296). All values represent mean ± SEM.

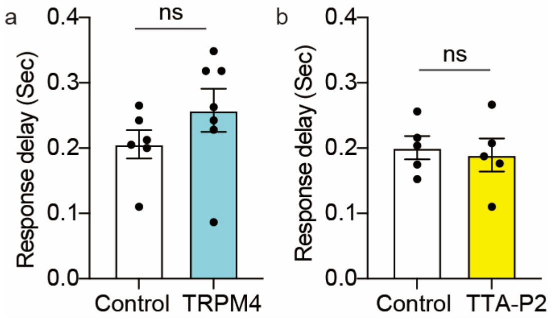

Supplementary Figure 10 | Quantification of response onset time after CRISPR knockdown of (a) TRPM4 (n=6 (control), 7 (TRPM4), Unpaired T-test, two-tailed, p=0.2332), and (b) after blocking T-type calcium channel (n= 5, Unpaired T-test, two-tailed, p=0.7296). All values represent mean ± SEM.

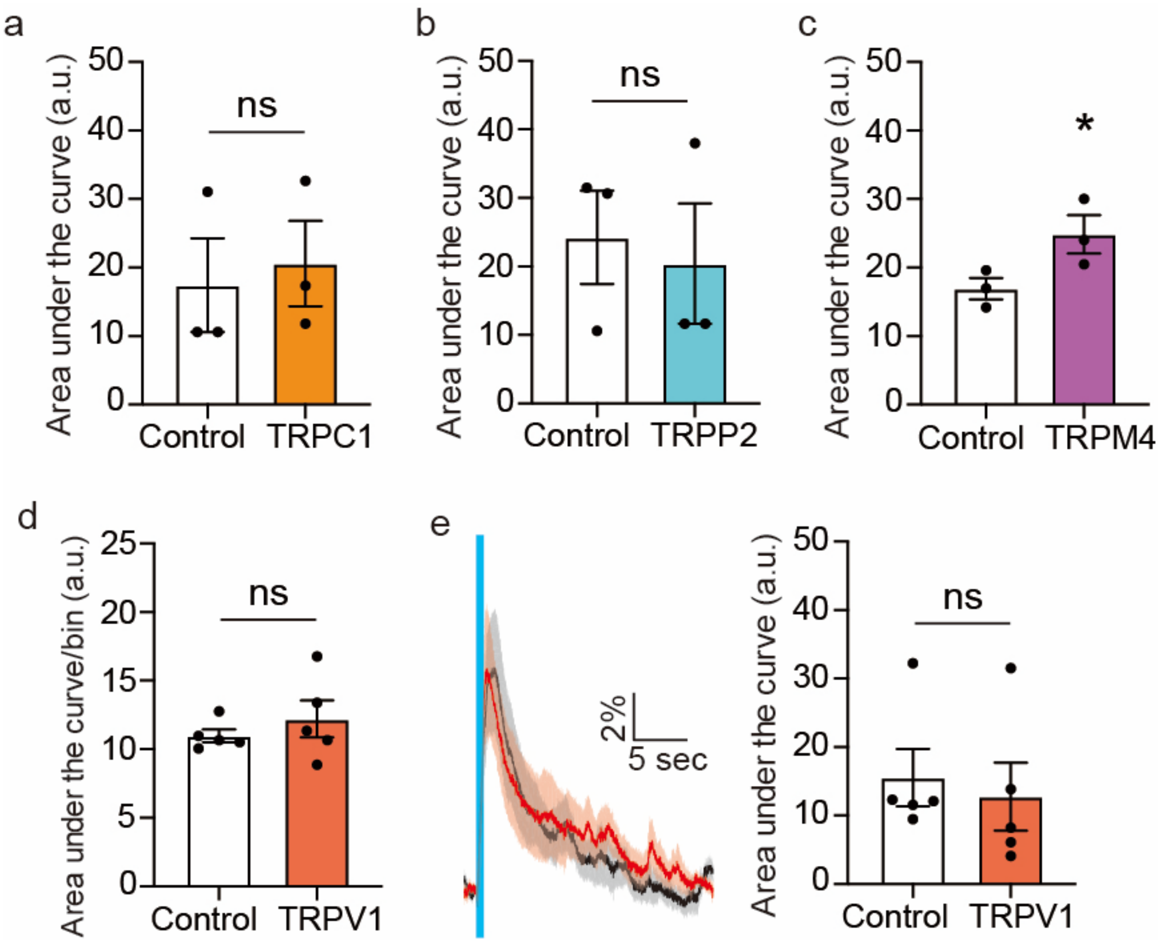

Supplementary Figure 11 | Overexpression of mechanosensitive channels. (a) Quantification of spontaneous activity after overexpressing TRPC1 (n= 3 independent experiments, Unpaired t-test, two-tailed, P=0.7491), (b) TRPP2 (n= 3 independent experiments, Unpaired t-test, two-tailed, P=0.7473), and (c) TRPM4 (n= 3 independent experiments, Unpaired t-test, two-tailed, P=0.0683). (d) Quantification of spontaneous activity after overexpressing TRPV1 (n= 5 independent experiments, Unpaired t-test, two-tailed, P=0.4191). (e) Calcium response to ultrasound after overexpressing TRPV1 (n= 5 independent experiments, Unpaired t-test, two-tailed, P=0.6801). All values represent mean ± SEM.

Supplementary Movie 1 | Calcium responses of neurons to ultrasound stimulation. Real time imaging of calcium

responses from GCaMP6f neurons to ultrasound stimulation. Ultrasound intensity at 15 W/cm^2 was applied for 500 ms 2 times with 20 sec inter-pulse interval.

Supplementary Table 1. sgRNAs for CRISPR knockdown of mechanosensitive channels

Target channel	sgRNA	PCR primers for seq. (For/Rev)
TRPM4	ACACAGTGGCCGGATCCGTG	caatgtgtctccccattg/ gaaaccctgtcttaaaaaagc
TRPM7	TCCAGGGTAATCTCCCCCCG	gatttgaactcaggacttcc/ gatggatctctatgttgcc
TRPP1	GAACTTGGCATACGGCATCG	cattagctcaacacgctg/ gagtatacccttacaacccac
TRPP2	CCAATGTGTACTACTACACT	ccaccttgctacacatttc/ cctgccattttagcactg
Piezo1	TGGGGCTGGAGTAGTTAGGG	cttcagctaagcagatgag/ ctttccaataattccctgacc
TRPC1	TCTTACAGGTGGGCTTACGG	cagagtggtagaacacttg/ ccacagtatgagaaggtaatc
Scramble	ACAACACGCCGACACGTCTA	

Supplementary Table 2. Predicted off target DNAs and their CFD score

Target channel	sgRNA	Off target DNA	Chrm..	Position	Mismatching	CFD score
TRPM4	ACACAGTGGCCGGATCCGTGNGG		n/a			
TRPM7	TCCAGGGTAATCTCCCCCCGNGG		n/a			
TRPP1	GAACTTGGCATACGGCATCGNGG	GAACTcGGCATAgGGCATCtAGG	chr7	58190249	3	0.086776859
		GAACgTGcCATACGGCATCcTGG	chrX	7697030	3	0.168791209
TRPP2	CCAATGTGTACTACTACACTNGG	CaAATGaGTACcACTACACTGGG	chr8	86046724	3	0.602870813
		gCtATGTGTcCTACTACACTAGG	chr7	129894326	3	0.214833759
		CCAAaaTGTACTACaACACTGGG	chr4	107776116	3	0.289473684
		CCAATGTGTACTACcACcgTAGG	chr4	116068603	3	0.006493506
		CaAATGTGTACTACctCACTTGG	chr5	122374594	3	0.010017531
		CaAATGTaTACTACcACACTTGG	chr5	133111346	3	0.198347108
		gCAATGTGTACTACTACAagAGG	chr15	7216283	3	0.069053708
		CCAATGTGTAaTgCcACACTGGG	chr6	113238793	3	0.13339921
		CCAcTGTGTACcgCTACACTTGG	chr14	102951205	3	0.218064342
		CCAATGTGTACcACTACAtgGGG	chr9	110447439	3	0.077161229
		CCAtTGTGTAgTACTACAtTGGG	chrX	61766440	3	0.07342657
		CCAcTtTGTACTACTACAtTTGG	chr11	25615191	3	0.108597285
		CaAATGTGTACcACcACACTGGG	chr11	65510726	3	0.187907786
		CaAATGTGTgCTACTACACTTGG	chr2	6718054	2	0.404040404
**Piezo1**	TGGGGCTGGAGTAGTTAGGGNGG	TGGGGCaGGAGTAGgTgGGGTGG	chr8	38306807	3	0.007720588
		TGGGGCaGGAGTAGgTAGGtGGG	chr12	37700661	3	0.030625
		TGGGGCTGaAGTgGTaAGGGAGG	chr12	86241822	3	0.381140599
		TGGGGagGGAGTAGTgAGGGAGG	chr3	84110027	3	0.18907563
		TGGGGCTGGAGaAGcaAGGGTGG	chr7	109584451	3	0.198347108
		TGGGGCaGGAGTAGgTAGGtGGG	chr7	120173029	3	0.030625

		TGGGGgTGGAGTAaTcAGGGAGG	chr7	139163261	3	0.25
		TGGGGCgGagGTAGTTAGGGTGG	chr1	39664346	3	0.210084034
		TGGGGaaGGAGTAGTTAGGaTGG	chr15	35582782	3	0.76171875
		TGaGGCTGGAGgAGaTAGGGAGG	chr15	101772220	3	0.217105263
		TGGGGCTGGAGcAGTgAGGcAGG	chr17	44247595	3	0.140543667
		TGaGGCTGGAGTAGgTgGGGGGG	chr10	26559790	3	0.006617647
		TGGGGCTGGAGaAGTgAGGcAGG	chr6	84393994	3	0.118681319
		TGGGGgTGGAGTtGgTAGGGTGG	chr6	98682092	3	0.0075
		TGGGGCTGcAcTAGTgAGGGAGG	chr6	110647213	3	0.079881657
		TGtGGCTGGAGTgGgTAGGGTGG	chr6	123922209	3	0.016304348
		TGGGGCTGGAGTctTTAGaGAGG	chr14	116081202	3	0.040100251
		TGGGGCTGGAGTgGgTAGGtGGG	chr9	36602467	3	0.022826087
		TGGGGgTGGAGaAGTTgGGGGGG	chr18	69502225	3	0.070588235
		TGGGGgTGGAGgAGTTgGGGTGG	chr11	116447021	3	0.044117647
**TRPC1**	TCTTACAGGTGGGCTTACGGNGG	TCTTcCAGGTGGGaTTACGtTGG	chr12	33176485	3	0.1225
		TCTTACAGcTGatCTTACGGAGG	chr3	139139588	3	0.150769231
		TCTTACAGGTatGCTTACaGAGG	chr16	59945876	3	0.274725275
		TCTaACAcGTGaGCTTACGGGGG	chr1	189833763	3	0.273504273
