# Supplementary material for: Focused ultrasound excites cortical neurons via mechanosensitive calcium accumulation and ion channel amplification
Source: Nat Commun. 2022 Jan 25;13:493. doi: 10.1038/s41467-022-28040-1 (PMC8789820; doi:10.1038/s41467-022-28040-1)
Supplement: Supplementary file 3 — Description of Additional Supplementary Files [file 41467_2022_28040_MOESM3_ESM.pdf]

Description of Additional Supplementary Files

Title: Supplementary Movie 1

Description: Real time imaging of calcium responses from GCaMP6f neurons to ultrasound stimulation. Ultrasound intensity at 15 W/cm2 was applied for 500 ms 2 times with 20 sec inter-pulse interval.
