# Supplementary material for: Focused ultrasound excites cortical neurons via mechanosensitive calcium accumulation and ion channel amplification
Source: Nat Commun. 2022 Jan 25;13:493. doi: 10.1038/s41467-022-28040-1 (PMC8789820; doi:10.1038/s41467-022-28040-1)
Supplement: Supplementary file 5 — Reporting Summary [file 41467_2022_28040_MOESM5_ESM.pdf]

nature research

Corresponding author(s): Mikhail Shapiro

Last updated by author(s): 12/4/2021

Reporting Summary

Nature Research wishes to improve the reproducibility of the work that we publish. This form provides structure for consistency and transparency in reporting. For further information on Nature Research policies, see our Editorial Policies and the Editorial Policy Checklist.

Statistics

For all statistical analyses, confirm that the following items are present in the figure legend, table legend, main text, or Methods section.

n/a	Confirmed	
☐	☒	The exact sample size (n) for each experimental group/condition, given as a discrete number and unit of measurement
☐	☒	A statement on whether measurements were taken from distinct samples or whether the same sample was measured repeatedly
☐	☒	The statistical test(s) used AND whether they are one- or two-sided<br>*Only common tests should be described solely by name; describe more complex techniques in the Methods section.*
☐	☒	A description of all covariates tested
☐	☒	A description of any assumptions or corrections, such as tests of normality and adjustment for multiple comparisons
☐	☒	A full description of the statistical parameters including central tendency (e.g. means) or other basic estimates (e.g. regression coefficient) AND variation (e.g. standard deviation) or associated estimates of uncertainty (e.g. confidence intervals)
☐	☒	For null hypothesis testing, the test statistic (e.g. F, t, r) with confidence intervals, effect sizes, degrees of freedom and P value noted<br>*Give P values as exact values whenever suitable.*
☒	☐	For Bayesian analysis, information on the choice of priors and Markov chain Monte Carlo settings
☒	☐	For hierarchical and complex designs, identification of the appropriate level for tests and full reporting of outcomes
☒	☐	Estimates of effect sizes (e.g. Cohen's d, Pearson's r), indicating how they were calculated

Our web collection on statistics for biologists contains articles on many of the points above.

Software and code

Policy information about availability of computer code

Data collection	Micro-Manager (version 1.4.15) was used for imaging and collecting calcium signals from cells. NeuroCa (Jang et al., 2015) was used for extracting calcium signals from each single cell. Density of DNA samples was measured using the NanoDrop 2000c software (version 1.5, Thermo Fisher Scientific). All custom code will be available upon request to authors.
Data analysis	We used MATLAB (version 2016b, Mathworks) , Excel (version 2104, Microsoft), ImageJ2 (version 2.3.0/1.53f, NIH) and Prism (version 8 and 9, Graphpad) for data and image analysis, statistic test, and plotting. TIDE (version 3.3.0) was used for quantification of CRISPR efficiency. Illustrations were made in Adobe Illustrator (version 25.4, Adobe).

For manuscripts utilizing custom algorithms or software that are central to the research but not yet described in published literature, software must be made available to editors and reviewers. We strongly encourage code deposition in a community repository (e.g. GitHub). See the Nature Research guidelines for submitting code & software for further information.

Data

Policy information about availability of data

All manuscripts must include a data availability statement. This statement should provide the following information, where applicable:

- Accession codes, unique identifiers, or web links for publicly available datasets
- A list of figures that have associated raw data
- A description of any restrictions on data availability

Source data is included with the manuscript. Raw image data is available upon reasonable request from the corresponding author.

Field-specific reporting

Please select the one below that is the best fit for your research. If you are not sure, read the appropriate sections before making your selection.

- [x] Life sciences
- [ ] Behavioural & social sciences
- [ ] Ecological, evolutionary & environmental sciences

For a reference copy of the document with all sections, see nature.com/documents/nr-reporting-summary-flat.pdf

Life sciences study design

All studies must disclose on these points even when the disclosure is negative.

Sample size	The numbers of biological and technical replicates were chosen based on preliminary experiments, so as to provide sufficient power for statistical comparison.
Data exclusions	no data were excluded
Replication	Replicates reported in the figures.
Randomization	All groups for cell imaging and ultrasound stimulation were randomized.
Blinding	No blinding was performed and was not needed because there was no subjective analysis.

Reporting for specific materials, systems and methods

We require information from authors about some types of materials, experimental systems and methods used in many studies. Here, indicate whether each material, system or method listed is relevant to your study. If you are not sure if a list item applies to your research, read the appropriate section before selecting a response.

Materials & experimental systems

n/a	Involved in the study	
[ ]	[x]	Antibodies
[ ]	[x]	Eukaryotic cell lines
[x]	[ ]	Palaeontology and archaeology
[ ]	[x]	Animals and other organisms
[x]	[ ]	Human research participants
[x]	[ ]	Clinical data
[x]	[ ]	Dual use research of concern

Methods

n/a	Involved in the study	
[x]	[ ]	ChIP-seq
[x]	[ ]	Flow cytometry
[x]	[ ]	MRI-based neuroimaging

Antibodies

Antibodies used	Anti beta-tubulin (1:500, Sigma, #T2200) was used as a neuron marker for all immunostaining. Alexa Fluo 488 Phalloidin (1:500, Thermo Fisher Scientific, #A12379) was used for actin staining shown in Fig2h. Anti-TRPC1 (1:200, Alomone Labs, #ACC-010), anti-TRPM4 (1:200, Alomone Labs, #ACC-044) and anti-TRPP2 (1:200, Alomone Labs, #T-155) were used for each channel staining shown in Fig 7a.
Validation	The validation tests performed by Sigma-aldrich (anti beta-tubulin), Thermo Fiser Scientific (Alexa fluo 488 Phalloidin), allomone labs (anti-TRPC1, TRPM4, TRPP2) including Immunocytochemistry, HPLC purity test, knockout and bioassay test.

Eukaryotic cell lines

Policy information about cell lines

Cell line source(s)	HEK293T cells were ordered from American Type Culture Collection(ATCC). Spiking HEK293T cells were gift from Adam E. Cohen.
Authentication	The cells were authenticated by ATCC by STR profiling.
Mycoplasma contamination	Micoplasma testing was not performed.
Commonly misidentified lines (See ICLAC register)	No commonly misidentified lines were used.

Animals and other organisms

Policy information about studies involving animals; ARRIVE guidelines recommended for reporting animal research

Laboratory animals	Female C57BL/6J mice, from which embryonic day 18 pups of both sexes were collected.
Wild animals	No wild animals were used.
Field-collected samples	No field collected samples were used.
Ethics oversight	Institutional Animal Care and Use Committee of the California Institute of Technology

Note that full information on the approval of the study protocol must also be provided in the manuscript.
