# Supplementary material for: Focused ultrasound excites cortical neurons via mechanosensitive calcium accumulation and ion channel amplification
Source: Nat Commun. 2022 Jan 25;13:493. doi: 10.1038/s41467-022-28040-1 (PMC8789820; doi:10.1038/s41467-022-28040-1)
Supplement: Supplementary file 6 — Source Data [file 41467_2022_28040_MOESM6_ESM.zip › Raw D_NatC_averaged/Note for data structure.rtf]

1. Each of m files are composed of several sub-m files, eg, fdata1, fdata2, fdata3...2. Each fdata is averaged delta-f data from a dish.3. First row in a fdata is time (sec).4. Second row in a fdata is GCaMP6f calcium signal.5. Time stamps for stimulation are: 19.3749000000000;39.3749000000000;59.349000000000;79.3749000000000;99.3749000000000 (5 stimulation)19.3749000000000;39.3749000000000;59.349000000000;79.3749000000000;99.3749000000000;119.3749000000000;139.3749000000000;159.3749000000000;179.3749000000000;199.3749000000000 (10 stimulation) 
