# Supplementary figures and images for: Focused ultrasound excites cortical neurons via mechanosensitive calcium accumulation and ion channel amplification
Source: Nat Commun. 2022 Jan 25;13:493. doi: 10.1038/s41467-022-28040-1 (PMC8789820; doi:10.1038/s41467-022-28040-1)

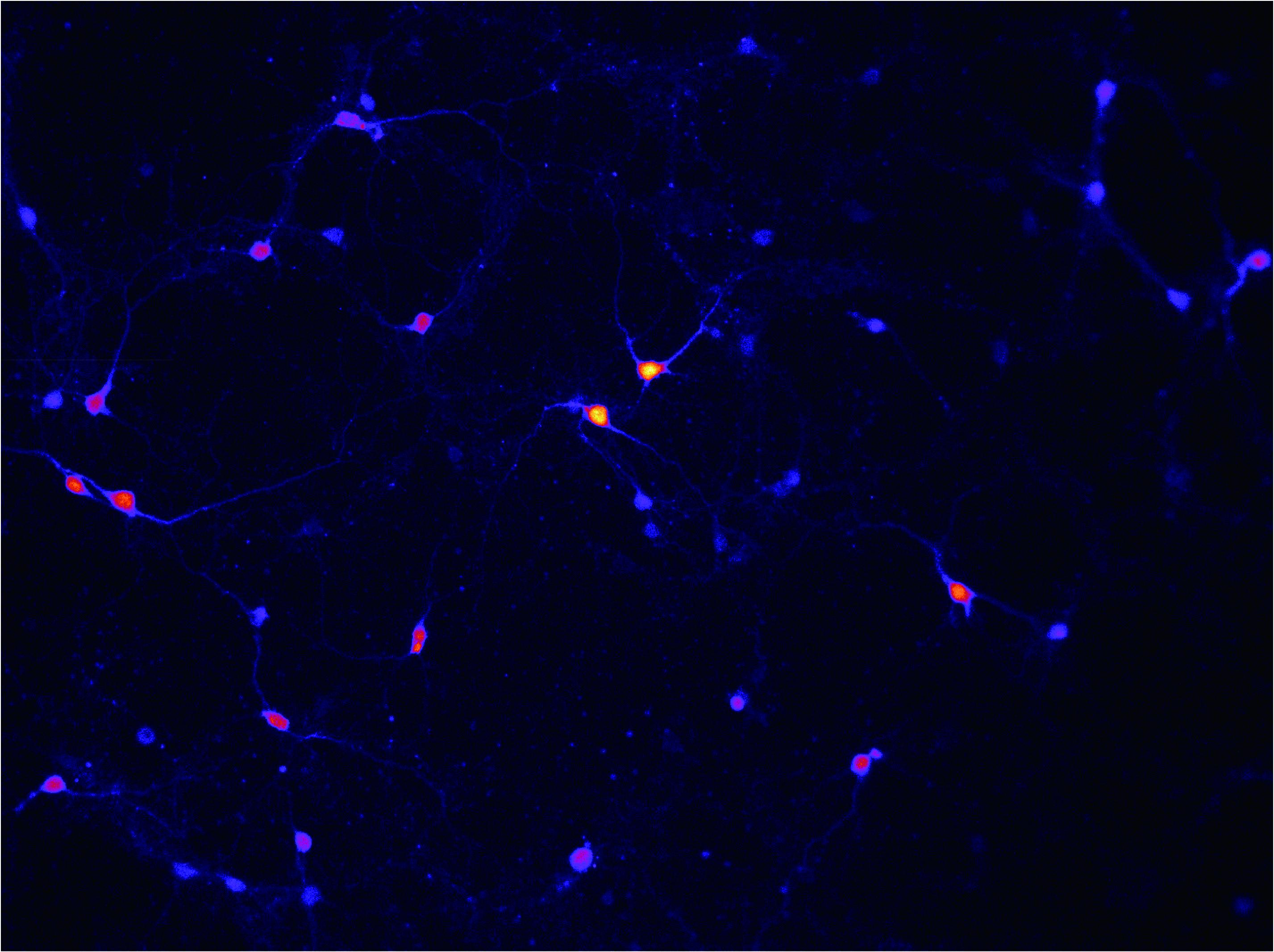

Supplement: Supplementary file 6 — Source Data [file 41467_2022_28040_MOESM6_ESM.zip › Raw D_NatC_averaged/FigS2/before.jpg]

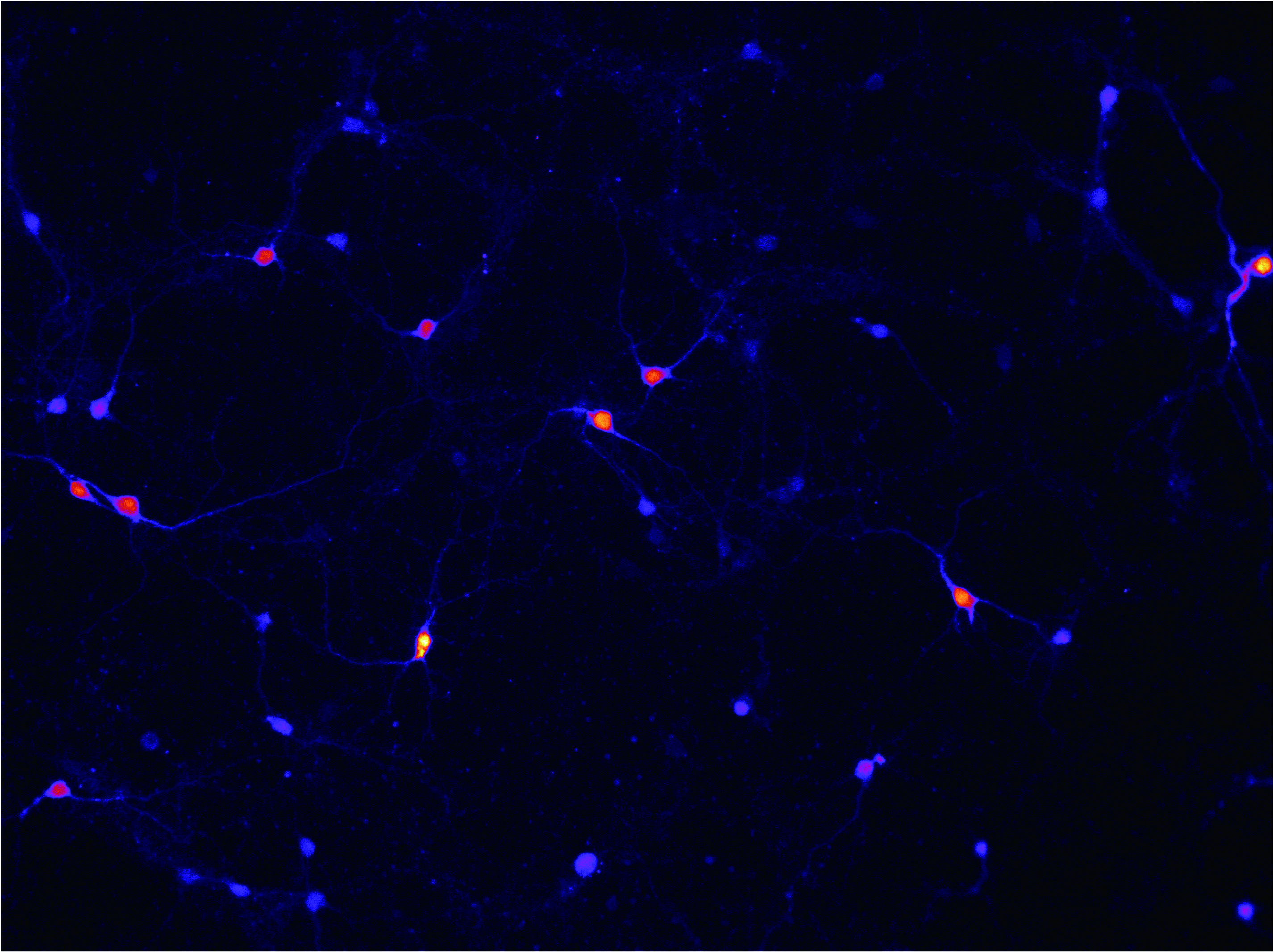

Supplement: Supplementary file 6 — Source Data [file 41467_2022_28040_MOESM6_ESM.zip › Raw D_NatC_averaged/FigS2/after.jpg]

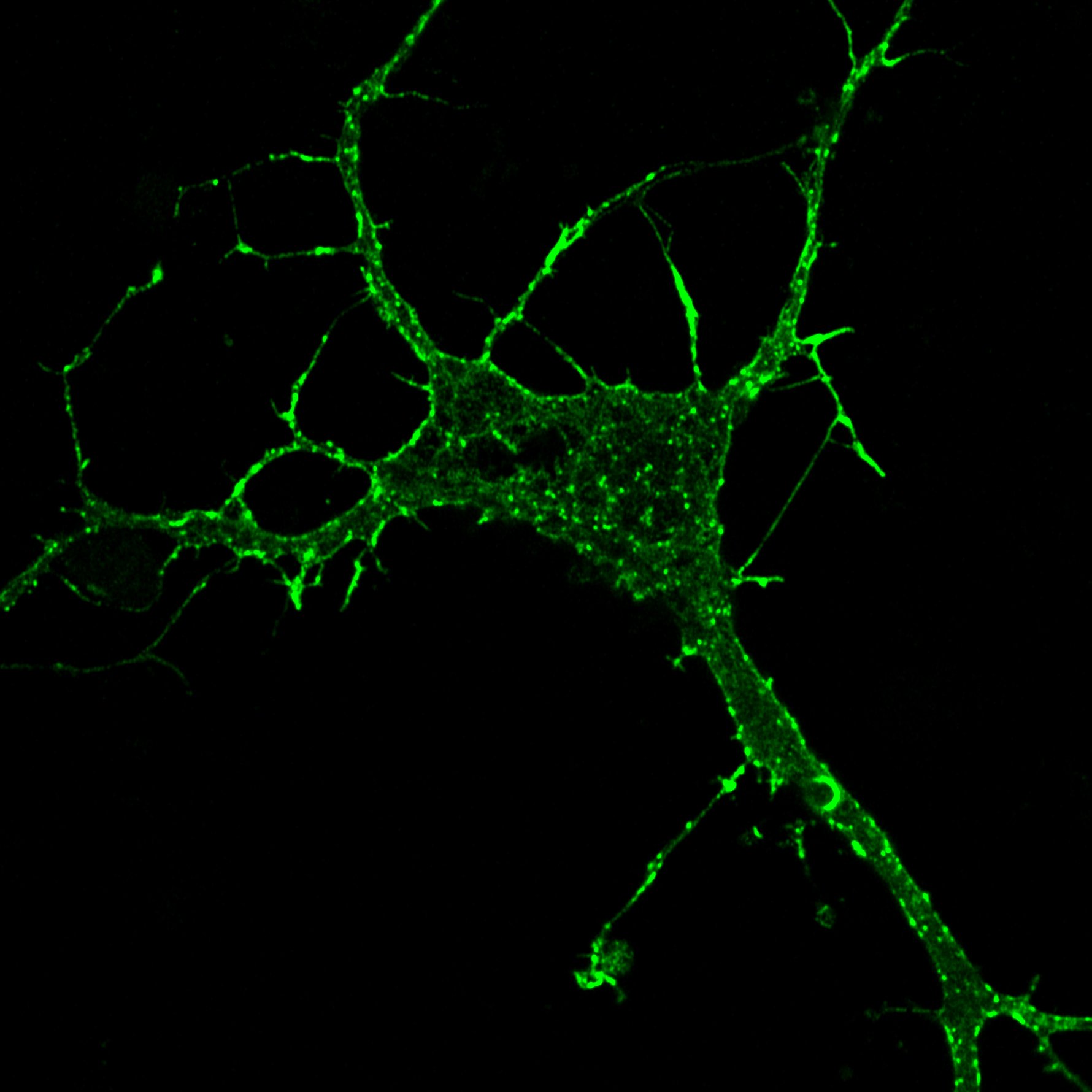

Supplement: Supplementary file 6 — Source Data [file 41467_2022_28040_MOESM6_ESM.zip › Raw D_NatC_averaged/Fig2/h-i_actin/Ctr_1_actin.jpg]

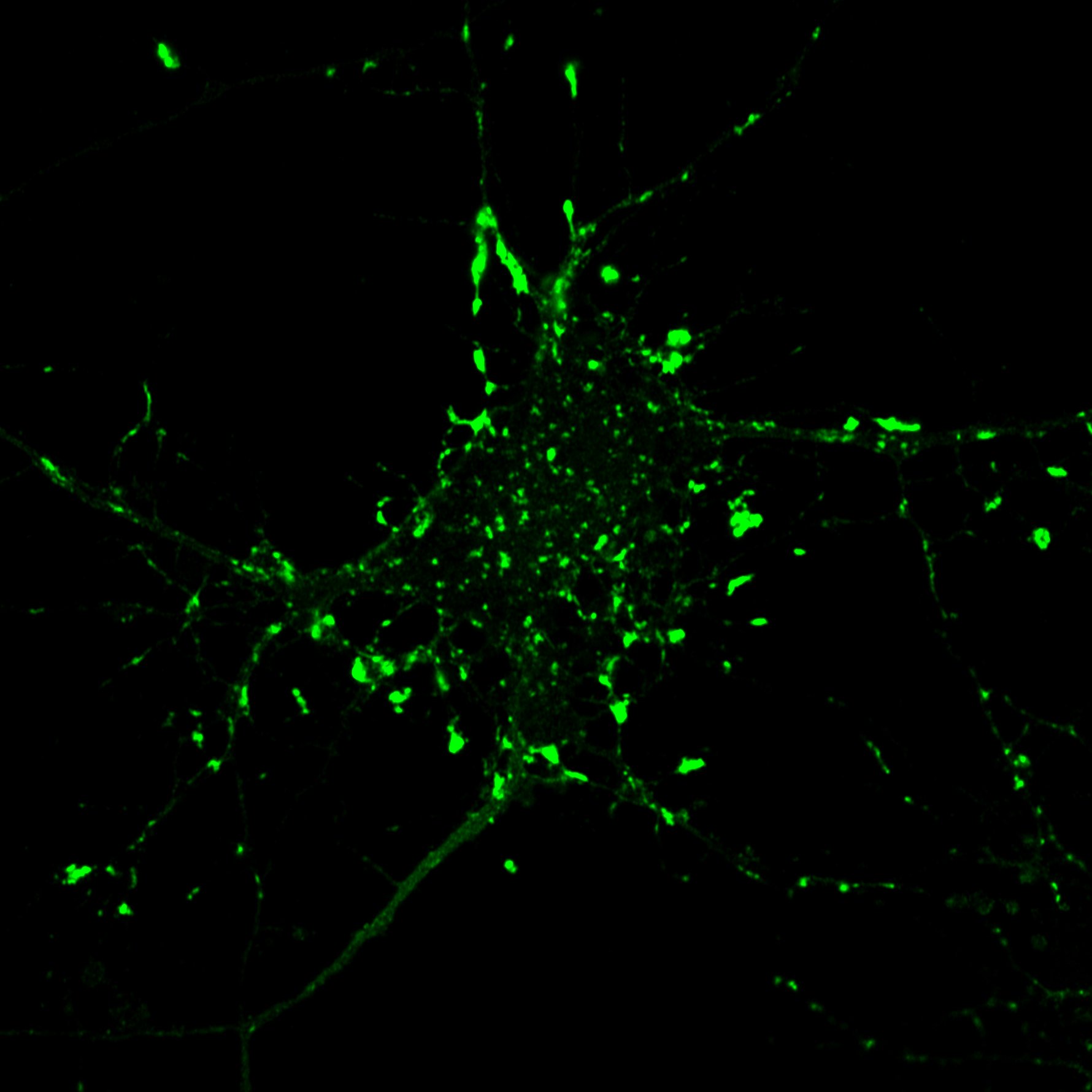

Supplement: Supplementary file 6 — Source Data [file 41467_2022_28040_MOESM6_ESM.zip › Raw D_NatC_averaged/Fig2/h-i_actin/1uM_4_actin.jpg]
